# Advocacy to reduce the risk of myopia

**Published:** 2019-05-13

**Authors:** Tim Fricke, Priya Morjaria, Sumrana Yasmin, Padmaja Sankaridurg

**Affiliations:** 1 Senior Research Fellow and Paediatric Optometrist: Brien Holden Vision Institute, Sydney, Australia.; 2 Research Fellow: Department of Clinical Research, London School of Hygiene and Tropical Medicine, International Centre for Eye Health, London, UK.; 3 Regional Director: South East Asia and Eastern Mediterranean, Brien Holden Vision Institute, Islamabad, Pakistan.; 4 Head: Global Myopia Centre, Brien Holden Vision Institute, Sydney, Australia.

**Encouraging and supporting the changes needed to prevent or delay the onset of myopia is complex. Co-ordinated advocacy is key.**

**Figure:**
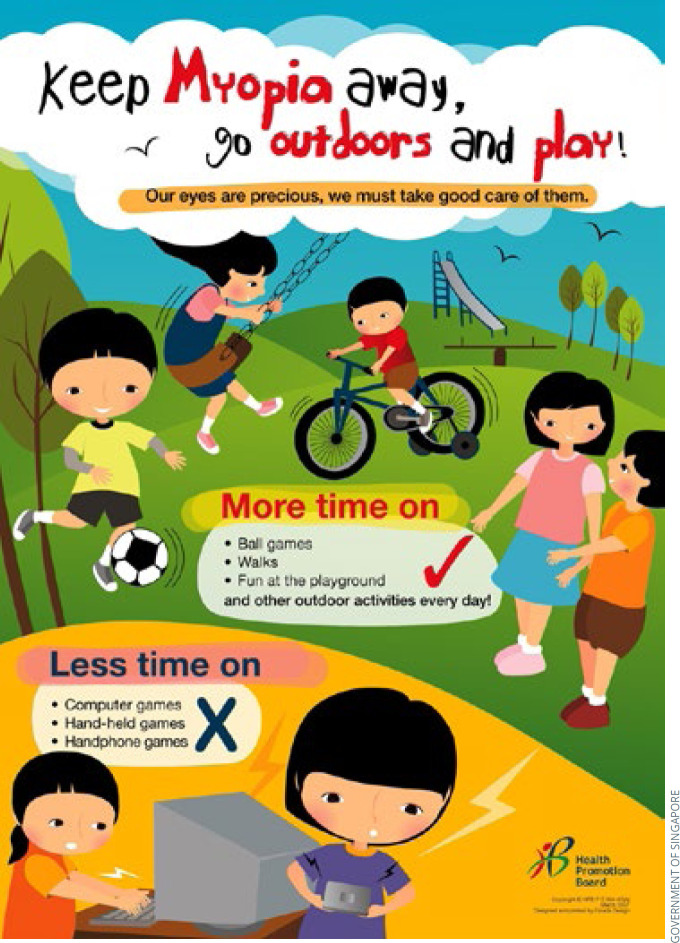
Posters by the government of Singapore encourage children to spend more time outdoors and reduce their screen time. SINGAPORE

The evidence for a global rise in the prevalence of myopia paints a worrying picture for the eye health of future generations. Although the causes are not completely understood, it is clear that myopia development and progression is multifactorial, and that environmental, lifestyle, and genetic factors are involved.^1^ The evidence is also clear that the myopia epidemic is real and that controlling it will require cross-sectoral efforts at collective and individual levels.

Although we have little ability to modify demographic risk factors such as ancestry, age and gender, it appears that myopia is also heavily influenced by modifiable risks such as near activity and outdoor time.

Modifying lifestyle factors, especially in young children, is likely to delay or prevent myopia onset. However, the influences on child behaviour are complex and vary with place and culture. Given these complexities, multi-dimensional approaches are needed to provide strategies that suit local situations and facilitate support from individuals, families, governments, health and educational bodies.

## Advocacy to minimise the risk factors for myopia onset

Advocacy – practical engagement through positive action and communication with the people and organisations who can make these changes – is essential. The suggestions below can be delivered by eye care practitioners, health bodies, governments, schools, etc. to individuals, families, schools, communities in order to modify the three risk factors with potential to influence myopia onset: an over-emphasis on education in children younger than 12 years old, lack of time outdoors, and too much time on near activities.

### Over-emphasis on education, particularly in children younger than 12 years of age

Advocacy aimed at:

- Encouraging balanced lifestyles for the purpose of eye health
- Improving ‘visual hygiene’ (sensible working distances, regular breaks from near tasks, good illumination)
- Making the educational environment ‘anti-myopic’ (e.g. bright classrooms).

### Lack of time outdoors

Advocacy aimed at:

- Encouraging balanced lifestyles for the purpose of eye health
- Town planning that encourages outdoor time
- Incorporation of outdoor time into education.

### Too much time on near activities

Advocacy aimed at:

- Encouraging balanced lifestyles for the purpose of eye health
- Limiting screen time.

We are facing a global myopia epidemic which requires combined efforts of researchers, parents, teachers, communities and governments to work together to reduce this burden that will affect us all economically via lost productivity, and will impact individuals' quality of life.

## References

[1] Lee Y-Y Lo C-T Sheu S-J, et al. What factors are associated with myopia in young adults? A survey study in Taiwan Military Conscripts. Investigative ophthalmology & visual science 2013;54(2):1026–33. 23322575 10.1167/iovs.12-10480

